# Surveillance of Mpox Cases in Mexico: Epidemiological Patterns During the 2022–2023 National Outbreak

**DOI:** 10.3390/diseases13090288

**Published:** 2025-09-01

**Authors:** Juan M. Bello-López, Dulce M. Razo Blanco-Hernández, Miguel Á. Loyola-Cruz, Clemente Cruz-Cruz, Oscar Sosa-Hernández, Nayeli G. Nieto-Velázquez, Georgina Victoria-Acosta, Adriana Jiménez, Laura Delgado-Balbuena, Luis G. Zárate-Sánchez, Paulina Carpinteyro-Espín, Enzo Vásquez-Jiménez, Adolfo López-Ornelas, Graciela Castro-Escarpulli, Araceli Rojas-Bernabé, María C. Tamayo-Ordóñez, Yahaira de J. Tamayo-Ordóñez, Francisco A. Tamayo-Ordóñez, Benjamín A. Ayil-Gutiérrez, Omar A. García-Hernández, Benito Hernández-Castellanos, Julio C. Castañeda-Ortega, Claudia C. Calzada-Mendoza, Emilio M. Durán-Manuel

**Affiliations:** 1 Hospital Juárez de México, Mexico City 07760, Mexico; 2 Sección de Estudios de Posgrado e Investigación, Escuela Superior de Medicina, Instituto Politécnico Nacional, Mexico City 11340, Mexico; 3 Hospital Nacional Homeopático, Hospitales Federales de Referencia, Mexico City 06800, Mexico; 4 Escuela Nacional de Ciencias Biológicas, Instituto Politécnico Nacional, Mexico City 11340, Mexico; 5 Facultad de Ciencias Químicas, Universidad Autónoma de Coahuila, Saltillo 25280, Mexico; 6 Centro de Biotecnología Genómica, Instituto Politécnico Nacional, Reynosa 88710, Mexico; 7 Facultad de Química, Universidad Autónoma del Carmen, Ciudad del Carmen 24180, Mexico; 8 SECIHTI-Centro de Biotecnología Genómica, Instituto Politécnico Nacional, Biotecnología Vegetal, Reynosa 88710, Mexico; 9 División de Investigación, Facultad de Medicina, Universidad Nacional Autónoma de México, Mexico City 54090, Mexico; 10 Facultad de Biología, Universidad Veracruzana, Xalapa 91090, Mexico

**Keywords:** mpox, epidemiology, Mexico, confirmed case, outbreak

## Abstract

Background: Mpox is an emerging zoonotic disease, caused by the monkeypox virus (MPXV). Since its discovery, it has been considered endemic in Central and West Africa. Mpox is of global significance as of May 2022, due to the report of simultaneous outbreaks in more than 70 countries where the disease was not endemic. The global spread of mpox has shown the importance of maintaining active surveillance for emerging zoonotic diseases, many of which can cross borders. Objective: The aim of this study was to analyse mpox cases and national incidence in Mexico related to the global outbreak. Methods: Epidemiological data (confirmed cases and incidence of MPXV infection) were obtained from the morbidity yearbook of the General Directorate of Epidemiology of the Mexican Ministry of Health. The information was analysed for the construction of epidemic curves, distribution of cases by age and sex and quartiles of geographical incidence. Results: A total of 4081 cumulative confirmed cases were recorded with a peak and national incidence of 1191 and 1.87, respectively, in September 2022. The distribution of cases by age and sex showed that males were more prevalent (above 95%) in the 25–44 years age group compared to females. Finally, geographical analysis showed that cosmopolitan and population-concentrated states had the highest incidence, clustered in the top quartile. The 2022 mpox outbreak in Mexico was consistent with other countries as reported in the international literature, with most cases occurring among exposed individuals in cosmopolitan cities. Conclusions: The need for active surveillance of emerging diseases, access to specific diagnostics and implementation of vaccination strategies is analysed and discussed.

## 1. Introduction

Mpox is an emerging zoonotic disease, caused by the monkeypox virus (MPXV) belonging to the family *Poxviridae* and the genus *Orthopoxvirus*. This virus has double-stranded DNA as its genetic material, to which also variola virus and vaccinia virus belong [1]. Although initially identified in 1958 in apes used for research in Denmark, it was not until 1970 that the first human case was reported in the Democratic Republic of Congo [2,3]. Since then, the disease has been considered endemic in several regions of Central and West Africa, mainly affecting communities living in close contact with wild animals [4]. Despite its African origin, mpox became globally relevant from May 2022, when simultaneous outbreaks began to be reported in more than 70 countries where the disease was not endemic [5,6].

In Europe, the epidemiological profile of Mpox has been characterized by a predominance of cases among men who have sex with men (MSM), with multiple clusters of community transmission reported across different countries during the 2022 outbreak. The European Multicenter Mpox Observational Cohort Study (MOSAIC) provided detailed insights into demographic, clinical, and epidemiological patterns of mpox in this region, highlighting variations in disease presentation and transmission dynamics [7].

This atypical spread represented a shift in the epidemiological dynamics of the virus and set off alarm bells for international health authorities. In countries such as the United States, Canada, the United Kingdom, Spain and other countries in Europe, multiple chains of transmission were identified that were not related to travel to endemic areas, which provided evidence of community transmission [8,9,10]. Transmission of MPXV occurs mainly through direct contact with an infected person’s body fluids, respiratory secretions, and skin or mucosal lesions [11]. In addition, indirect transmission through contaminated objects, such as contaminated clothing, bed linen, towels or utensils that have been in contact with contaminated fluids, has been documented [12]. Although MPXV was initially not considered a sexually transmitted infection (STI), recent evidence supports its classification as an STI, particularly for clade IIb [13,14,15]. Symptoms of mpox usually begin with fever, severe headache, lymphadenopathy, myalgia, and general fatigue. Subsequently, skin eruptions appear, characteristic of a progression from macules and papules to vesicles, pustules and finally crusts. Unlike other exanthematous diseases, generalized lymphadenopathy is a hallmark of mpox [16]. Regarding immune responses, adaptive immunity plays a critical role in the control of MPXV infection, with both B and T cells contributing to viral clearance. Recent studies have demonstrated robust CD4+ and CD8+ T cell responses in individuals infected with MPXV and in those previously vaccinated with vaccinia virus, suggesting cross-reactive cellular immunity [17,18]. The clinical course in most cases is self-limiting, lasting between two to four weeks, although complications such as secondary infections, encephalitis or pneumonia may occur in certain patients [19]. Currently, two clades of MPXV strains are recognized, depending on their geographic location and virulence. Clade I strains from Central Africa have been shown to be more virulent than clade II strains from West Africa, as case fatality rates of up to 10% have been observed for clade I [20].

The severity of the clinical picture may be increased in immunocompromised individuals, infants, older adults and pregnant women, which may result in death. In these groups, early medical care and surveillance are critical to reduce mortality [21]. Vaccination is a key tool in disease prevention and control. Third-generation vaccines based on non-replicating vaccinia virus, and second-generation vaccines based on replicating virus, have been shown to be effective in preventing MPXV infection, especially if administered before or shortly after exposure to the virus. These vaccines are especially recommended for people with risky sexual practices, close contacts of confirmed cases and health professionals working in high-risk settings [22].

The response to the mpox emergency has also involved the implementation of strategies of epidemiological surveillance, isolation of suspected and confirmed cases, contact tracing, community education and information, and the use of personal protective equipment (PPE) in clinical settings [23]. The importance of addressing the stigma associated with this disease has also been emphasized, particularly in LGBTQ+ communities, where a disproportionate concentration of cases has been observed during the 2022 outbreak, but should not be mistakenly considered as solely responsible for transmission [24,25]. Studies have shown that, despite the implementation of containment measures, there is still a need for active epidemiological surveillance to understand the dynamics and distribution of the disease in the population.

This need is particularly relevant in those countries that have been affected by this global outbreak, including Mexico. The global spread of mpox has highlighted the importance of considering emerging zoonotic diseases as a priority public health issue, to the point of being included as a notifiable disease. Mpox represents a paradigmatic example of pathologies capable of rapidly crossing geographical borders, facilitated by phenomena such as globalization, international travel and close human–animal contact [20]. The aim of this work was to analyze in Mexican territory the cases and national incidence of mpox related to the global outbreak during 2022. The need for active surveillance of emerging diseases, access to specific diagnostics and implementation of vaccination strategies is analysed and discussed.

## 2. Materials and Methods

### 2.1. Cases and Incidence of Mpox

This work reports the confirmed cases and incidence of mpox during the period 2022 and 2023 in Mexican territory, issued by the National Epidemiological Surveillance System. Epidemiological data for 2024 is not yet available on the website consulted. The Mexican regulation establishes mandatory reporting of confirmed cases of mpox in the 32 states that make up the Mexican territory.

### 2.2. Operational Definitions of Mpox

The classification of cases followed the definitions from the “Standardized Procedures Manual for Epidemiological Surveillance of mpox, version 2.0”, issued by the General Directorate of Epidemiology of the Ministry of Health [16].

#### 2.2.1. Probable Case of Mpox

Any person presenting one or more skin or mucosal lesions of macular, papular, vesicular, pustular and/or crusting type without a clinical diagnosis and presenting one or more of the following symptoms: fever, myalgia, headache, lymphadenopathy, asthenia, arthralgia and back pain. In the case of immunocompromised persons, the presence of the above-mentioned skin or mucosal lesions without the manifestation of signs or symptoms is considered a probable case.

#### 2.2.2. Laboratory Confirmed Case of Mpox

This is a probable case that presents a positive result for MPXV analysed by the Instituto de Diagnóstico y Referencia Epidemiológicos (Institute of Diagnosis and Epidemiological Reference, InDRE, by its acronym in Spanish) or a laboratory verified and trained for this diagnosis. This is in accordance with the Guidelines for laboratory surveillance of Mpox version 2.0, issued by the Undersecretary of Health Policies and Population Welfare [25]. Briefly, the probable case is submitted for sampling under strict biosafety to initiate the MPXV detection algorithm according to Li et al., which consists of a first real-time PCR reaction (species-specific) and a second and third PCR reaction for virus classification by clade II and clade I, respectively [26]. In some cases, PCR differentiation of clade IIb can be performed. For general epidemiological purposes, all confirmed cases (without information about Clade assignment) were deposited in the morbidity yearbook (1984–2023) of the General Directorate of Epidemiology (GDE).

#### 2.2.3. Case Confirmed by Clinical Epidemiological Association

This is a probable case that does not have a sample and that has documented epidemiological association in the 21 days prior to the onset of symptoms with at least one laboratory-confirmed case. In contrast, a probable case is confirmed if it presents an inconclusive laboratory result and for some reason it is not possible to take a second sample for analysis in accordance with the diagnostic algorithm and if the epidemiological association with a laboratory-confirmed case has been documented.

#### 2.2.4. Discarded Case

Probable cases with a negative result for MPXV tested by InDRE or by authorised and verified laboratories, without requiring clinical or epidemiological association.

#### 2.2.5. Definition of Contact

Any person who has had one or more of the following exposures to a probable or confirmed case within the past 21 days: direct skin-to-skin and/or sexual contact, inhalation of respiratory aerosols from infected persons, contact with material from skin or mucosal lesions such as crusts, contact with fomites or contaminated material, clothing from a confirmed or probable case, bedding and personal utensils.

### 2.3. Data Collection

Data collection was carried out respecting the principles of confidentiality and discretion outlined in the Federal Law on Transparency and Access to Public Information. Epidemiological data was taken from the morbidity yearbook (1984–2023) of the GDE, available at https://epidemiologia.salud.gob.mx/anuario/html/index.html (accessed 21 March 2025). The information reported on this platform has been previously generated and analysed by the Ministry of Health through its website www.sinave.gob.mx (accessed 21 March 2025). For this study, we included all confirmed cases of mpox (laboratory-confirmed or confirmed by clinical–epidemiological association) reported in the national morbidity yearbook for 2022–2023. No ethical approval was obtained for the use of epidemiological information on mpox in Mexico.

### 2.4. Analysis of Confirmed Cases and Incidence of Mpox

The confirmed cases and incidence of mpox (per 100,000 habitants) reported by the GDE were analysed by month, year, sex, age group and incidence for the 32 states of the Mexican Republic during the period 2022 (May/December)–2023 (January/December). With this information, epidemic curves of cases and incidence of MPXV infection were constructed and visualized using Microsoft Excel (Microsoft Corporation, Redmond, WA, USA), which was also employed to analyse cumulative cases by age group and sex. In addition, an analysis describing the cumulative cases by age group and sex was performed. Finally, an analysis of incidence by geographical distribution was carried out, considering the 32 states of the Mexican Republic and grouped into quartiles, with quartiles 4 (Q_4_) and 1 (Q_1_) having the highest and lowest incidence, respectively. Once the 32 states were stratified into quartiles by incidence, incidence means were estimated and the temporal behavior of each quartile during the study period was evaluated to confirm the states with the highest incidence of mpox.

## 3. Results

### 3.1. Confirmed Cases and Incidence of Mpox

**Figure 1:**
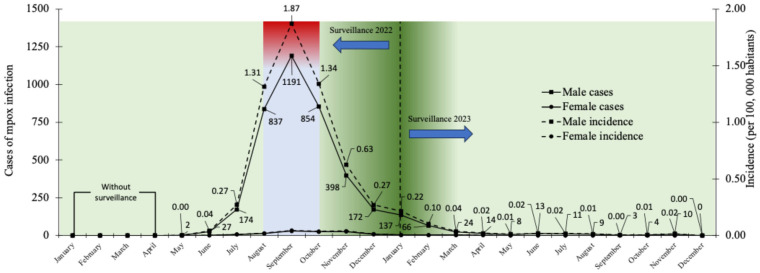
Temporal epidemic curve of confirmed cases and incidence (per 100,000 habitants) by sex of mpox in Mexico during 2022 and 2023.

During the study period, 4081 cumulative confirmed cases were recorded, with 3952 (96.84%) and 129 (3.16%) cases for males and females, respectively. The monthly average number of cases was 312 and 26 for 2022 and 2023 with cumulative cases for these same years of 3769 and 312. This represents a 91.7% decrease in cases for both sexes, which translates into an absolute reduction of 3457 cases by 2023. It is important to note that active surveillance for mpox was conducted from May 2022 (Figure 1). A progressive increase in cases and incidence was identified, with a peak in September 2022 in the male sex of 1191 and 1.87, respectively. In contrast, the female sex showed the lowest number of cases and incidence, with a maximum peak of both indicators in the same month as for the male sex of 30 and 0.044. Finally, from March 2023 onwards, cases and incidence remained stable until December of the same year. Figure 1 shows the temporal epidemic curve of confirmed cases and incidence (per 100,000 habitants) by sex of mpox in Mexico during 2022 and 2023.

### 3.2. Distribution of Mpox Cases by Age Group and Sex

**Figure 2:**
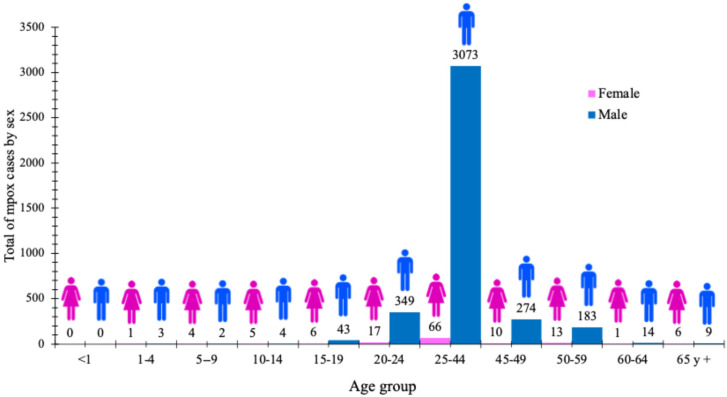
Distribution of cumulative confirmed cases of mpox in Mexican territory by age group and sex during 2022 and 2023.

The analysis of confirmed cases by age group and sex revealed that in ten of the eleven age groups analysed, at least three confirmed mpox cases fell into the pediatric male age group. This trend was identified in the following age groups of this sex. Conversely, in the <1 year age group, no cases were recorded for both sexes. The male sex revealed a predominance of accumulated cases with a total of 3952, in contrast to females, where 129 confirmed cases were observed. This corresponds to a case ratio of 97% and 95.2% for 2022 and 2023, respectively, compared to females. The distribution of cases by age group showed a maximum peak of 3071 cases in men aged 25 to 44 years old, followed by the groups aged 20 to 24, 45 to 49, and 50 to 59 years old for a total of 806 cases. The group that concentrated the most cases accounted for 77.7% of all male cases in the age groups analysed. Finally, the peak number of cases in females was 66 in the 25–44 age group. The distribution of cumulative confirmed cases of mpox in Mexican territory by age group and sex during 2022 and 2023 is shown in Figure 2.

### 3.3. Geographical Distribution of the Incidence of Mpox in Mexico

In general, the states with the highest incidence of cases were those located in the center, western Pacific coast and south of the country, bordering countries such as Belize and Guatemala. Furthermore, based on the average state incidences during the study period, states were classified into quartiles (Q_1_–Q_4_).

**Figure 3:**
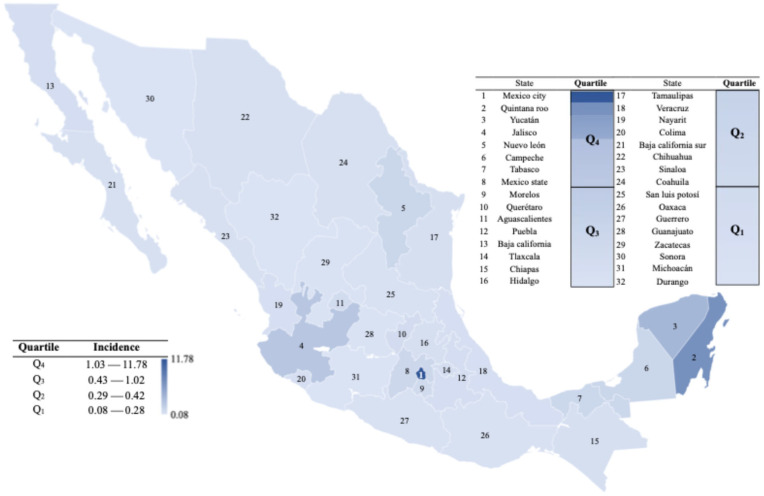
Geographical and quartile distribution of mpox incidence (per 100,000 habitants) in Mexico during 2022–2023.

The quartile analysis showed that the highest incidences were concentrated in Q_4_ represented by eight of the 32 states, such as Mexico City (11.78), Quintana Roo (6.84), Yucatán (3.68), Jalisco (2.39), Nuevo León (1.19), Campeche (1.16), Tabasco (1.09), and Mexico State (1.03). Interestingly, most of the states that made up Q3 are adjacent to Mexico City, the state with the highest incidence of cases (Figure 3). Conversely, the lowest incidence values were grouped in the remaining three quartiles (Q1, Q2, and Q3), with Durango being the state with the lowest incidence of cases, and the one that showed at most one case in the months of September and October 2022 and January 2023. Figure 3 shows the geographical and quartile distribution of mpox incidence (per 100,000 habitants) in Mexico during 2022–2023.

### 3.4. Monthly Behavior of the National Incidence of Mpox According to Quartiles

**Figure 4:**
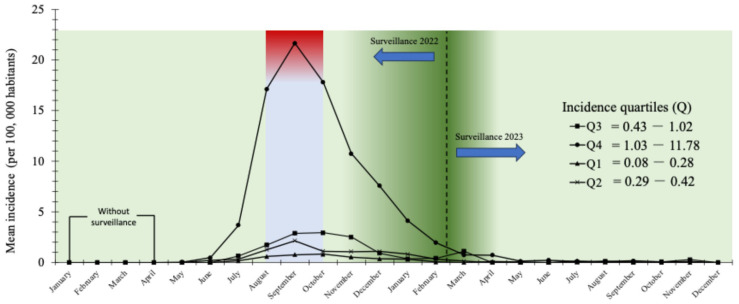
Monthly behaviour of the average incidence (per 100,000 habitants) in Mexican territory of mpox classified by quartiles (Q1, Q2, Q3, and Q4) during 2022–2023.

In order to determine whether the highest incidence of cases was concentrated in those states that made up Q4 (Mexico City, Quintana Roo, Yucatán, Jalisco, Nuevo León, Campeche, Tabasco, and Mexico State), an analysis of the behavior of monthly incidence by quartiles was carried out with emphasis on Q4 (Figure 4). The results revealed a progressive increase from May 2022 (start of surveillance of mpox cases) of 0.034, with a maximum peak in September of 21.67 and a gradual decrease until May 2023, when the incidence of this quartile stabilized. This behavior was similar for the remaining quartiles where Q3 outperformed Q2 with a factor of 1.65, and the latter was 2.19 times higher than Q1. These results confirm that Q4 concentrates the states with the highest incidence of mpox cases. Figure 4 shows the monthly behavior of the average incidence (per 100,000 habitants) in Mexican territory of mpox classified by quartiles (Q1, Q2, Q3, and Q4).

## 4. Discussion

Emerging zoonotic diseases represent one of the greatest challenges for public health worldwide, due to their ability to cross species barriers and spread among human populations [27]. Pathogens that had been geographically limited due to region-specific climatic conditions have acquired new transmission routes that have been facilitated by globalization, climate change, urbanization, human mobility and others [28]. Recent experiences with emerging zoonotic diseases have shown how these pathogens can spread rapidly across countries and pose significant challenges to public health systems [29]. Mpox is a clear example of this dynamic, a zoonosis previously confined to regions of Central and West Africa, and which in 2022 was suddenly identified in more than 70 countries, including Mexico [30]. From May 2022 onwards, case detection began in high-income countries with no endemic history, demonstrating the vulnerability of countries with well-established health and epidemiological surveillance systems [31]. In this context, the case of Mexico during the outbreak that started in May 2022 evidenced the impact of the robust epidemiological surveillance system at the national level as a necessary tool to contain the spread of the disease. As shown in Figure 1, after a sustained increase in mpox cases, a peak was reached in September 2022, and after the implementation of active epidemiological surveillance, an impact on the reduction in cases was observed from October of the same year with a sustained decrease until reaching minimum levels in 2023. This pattern of behavior coincides with what has been reported in various epidemiological and molecular investigations. For example, an epidemiological week-by-week study of mpox Clade II cases in the United States revealed a peak of more than 3000 cases in week 30, corresponding to July 2022, with a progressive decline from August onwards [32].

In contrast to what was reported in this study, where a maximum of 1221 cases were reported, which was below the number reported by Tuttle et al., it must be recognized that there may be an under-reporting of cases that could limit national epidemiological surveillance. In addition, the operational definition used during the outbreak, which included generalized lymphadenopathy as a differentiating feature from chickenpox (as described in the Standardized Procedures Manual for Epidemiological Surveillance of Mpox, version 2.0), may not always reliably distinguish these infections, potentially contributing to misclassification or underreporting. Furthermore, in immunocompromised individuals, the clinical presentation of Mpox and chickenpox may be atypical, including absence or lower intensity of lymphadenopathy, further limiting the usefulness of this sign as a differentiating criterion. Our study has several limitations that should be acknowledged. First, the analysis was based on secondary data obtained from the national epidemiological surveillance system, which depends on the completeness and accuracy of case reporting by local health authorities. Second, underreporting is a potential issue, particularly in rural or marginalized areas where access to diagnostic services may be limited and risk perception is lower. Third, the operational definition applied during the outbreak, which included generalized lymphadenopathy as a differentiating criterion, may not reliably distinguish mpox from other exanthematous diseases such as chickenpox, especially in immunocompromised individuals. Finally, no molecular or clinical data were available to correlate disease severity or clade differences, which could provide further insights into the epidemiological patterns observed.

Nonetheless, in a paper by Scarpa et al. through a phylogenetic reconstruction study, two main findings were identified: first, a cumulative peak of more than 30,000 cases in August 2022 in countries of the Americas Region, European Region, African Region, and Western Pacific Region with a gradual reduction in cases from September of the same year [33]. The second important finding was that phylogenetic reconstruction analysis of MPXV identified that this virus has low evolutionary rates suggesting little likelihood of variant development. From a demographic point of view, 96.8% of the cases corresponded to men, with the 25–44 age group being the most affected. These findings are consistent with those observed in several countries, such as the UK, Spain, Canada, and Brazil [34,35,36,37]. According to these works, the majority of the affected population is overrepresented by men who have sex with men (MSM), which could translate into specific chains of sex-specific transmission, which has been widely discussed in previous works. Heskin et al. and Thornhill et al. have shown that more than 95% of mpox cases worldwide have occurred in men, many with a recent history of sexual contact with other men [9,38].

In the specific case of Mexico, data from the General Directorate of Epidemiology revealed that a significant proportion of confirmed cases had co-infection with the human immunodeficiency virus (HIV) and other sexually transmitted diseases, which confirms that the affected populations are highly vulnerable and that dual Mpox-HIV screening in clinics for people with sexually transmitted infections is important [39]. In addition, the impact of social stigmatization must be considered, as fear of discrimination may prevent people at risk from seeking timely care, which is also reflected in underreporting of cases. An important aspect of mpox transmission is related to cosmopolitan and/or tourist cities. As shown in Figure 3, there is a concentrated pattern of cases in central, southern and southeastern Mexico, with Mexico City, Quintana Roo, Yucatán and Jalisco accounting for the majority of cases (Q4), coinciding with previous studies that have shown that those regions with high population density, intense mobility, and high tourist activity are where the highest number of cases were detected [10,40,41]. Confirmation of areas of high disease transmission is of utmost importance to gather or focus control efforts in these areas as they could be considered potential foci of mpox dissemination. The quartile analysis depicted in Figure 4 confirms this trend for the states in the highest incidence quartile (Q4), being the states with the highest concentration of cases and representing the abrupt peak in September 2022, while the lower quartiles exhibited much lower incidence peaks.

This marked difference across Mexico’s territory suggests that urban characteristics, international connectivity such as the presence of airports, and healthcare infrastructure directly influence the speed of spread and, consequently, the need for disease containment. As mentioned above, this markedly geographic behavior shows the need to apply regionally differentiated epidemiological surveillance models, as not all states require the same epidemiological strategy. For example, Mexico City has specialized laboratories, access to diagnostic tests and rapid communication channels that could facilitate early detection, while other regions may require more complex and sometimes impossible to implement strategies. Undoubtedly one of the best strategies that serve the function of disease containment are those that are immediately adopted by first world countries, such as vaccination.

Countries such as the United States and Canada deployed almost immediate vaccination campaigns with the JYNNEOS vaccine in key populations [42,43,44]. In contrast, Mexico did not have a national vaccination strategy in place until 12 September 2024, two years after the first peak of the health emergency [45]. However, only the health registration for its application had been authorized by the Comisión Federal para la Protección contra Riesgos Sanitarios (Federal Commission for Protection against Health Risks, COFEPRIS, by its acronym in Spanish). This approval was granted after a technical-scientific evaluation process that concluded that the vaccine met the requirements of quality, safety and efficacy, so that, with this authorization, the vaccine could be legally marketed and used in Mexico, although its application is currently subject to the strategies determined by Mexican health agencies. Access to vaccines in low- and middle-income countries during health emergencies shows the need for national and international strategies that allow timely and equitable action with vaccines and/or treatments [46,47]. Such is the immediate need for access to vaccines that on 14 August 2024, the WHO reclassified mpox as a disease under intensive epidemiological surveillance due to a new outbreak, prompting international authorities to again strengthen surveillance and control measures [48]. This highlights the latent risk of new outbreaks in countries with socio-economic characteristics that could face new health emergencies.

## 5. Conclusions

The mpox outbreak highlights the need to strengthen epidemiological surveillance of emerging zoonotic diseases in Mexico. Initiatives such as the inclusion of mpox in the list of notifiable diseases are important steps in modern epidemiology, but they must be accompanied by public health strategies targeted to specific populations, scientific research, and training of healthcare personnel to prepare for the management of this disease.

## Data Availability

The data that support the findings of this study are openly available in Mendeley Data at https://data.mendeley.com/datasets/478wjdn2hj/1 (accessed on 16 May 2025).

## Abbreviations

MPXV	Monkeypox virus
InDRE	Institute of Diagnosis and Epidemiological Reference
COFEPRIS	Federal Commission for Protection against Health Risk
HIV	Human immunodeficiency virus
WHO	World Health Organization

## References

[1] Moore M.J., Rathish B., Zahra F. (2025). Mpox (Monkeypox). StatPearls.

[2] Cho C.T., Wenner H.A. (1973). Monkeypox virus. Bacteriol. Rev..

[3] Ladnyj I.D., Ziegler P., Kima E. (1972). A human infection caused by monkeypox virus in Basankusu Territory, Democratic Republic of the Congo. Bull. World Health Organ..

[4] Khodakevich L., Jezek Z., Messinger D. (1988). Monkeypox virus: Ecology and public health significance. Bull. World Health Organ..

[5] Laurenson-Schafer H., Sklenovská N., Hoxha A., Kerr S.M., Ndumbi P., Fitzner J., Almiron M., de Sousa L.A., Briand S., Cenciarelli O. (2023). Description of the first global outbreak of mpox: An analysis of global surveillance data. Lancet Glob. Health.

[6] World Health Organization 2024–2025 Mpox (Monkeypox) Outbreak: Global Trends. https://worldhealthorg.shinyapps.io/mpx_global/.

[7] Pesonel E., Laouénan C., Guiraud L., Bourner J., Hoffmann I., Molino D., Tardivon C., Bachelet D., Mentré F., Amstutz A. (2025). Mpox ObServAtIonal Cohort (MOSAIC) Study Group (2025). Clinical Characterization and Outcomes of Human Clade IIb Mpox Virus Disease: A European Multicenter Mpox Observational Cohort Study (MOSAIC). Clin. Infect. Dis..

[8] Vivancos R., Anderson C., Blomquist P., Balasegaram S., Bell A., Bishop L., Brown C.S., Chow Y., Edeghere O., Florence I. (2022). Community transmission of monkeypox in the United Kingdom, April to May 2022. Eurosurveillance.

[9] Heskin J., Belfield A., Milne C., Brown N., Walters Y., Scott C., Bracchi M., Moore L.S.P., Mughal N., Rampling T. (2022). Transmission of monkeypox virus through sexual contact—A novel route of infection. J. Infect..

[10] Martínez J.I., Montalbán E.G., Bueno S.J., Martínez F.M., Juliá A.N., Díaz J.S., Marín N.G., Deorador E.C., Forte A.N., García M.A. (2022). Monkeypox outbreak predominantly affecting men who have sex with men, Madrid, Spain, 26 April to 16 June 2022. Eurosurveillance.

[11] Mitjà O., Ogoina D., Titanji B.K., Galvan C., Muyembe J.J., Marks M., Orkin C.M. (2023). Monkeypox. Lancet.

[12] Venkatesan P. (2022). Monkeypox transmission—What we know so far. Lancet Respir. Med..

[13] Rizk J.G., Lippi G., Henry B.M., Forthal D.N., Rizk Y. (2022). Prevention and treatment of monkeypox. Drugs.

[14] Allan-Blitz L.T., Klausner J.D. (2023). Current Evidence Demonstrates That Monkeypox is a Sexually Transmitted Infection. Sex. Transm. Dis..

[15] Allan-Blitz L.T., Gandhi M., Adamson P., Park I., Bolan G., Klausner J.D. (2023). A Position Statement on Mpox as a Sexually Transmitted Disease. Clin. Infect. Dis..

[16] Secretaría de Salud (2022). Dirección General de Epidemiología. Manual de Procedimientos Estandarizados para la Vigilancia Epidemiológica de Viruela Símica. Versión 2.0. Ciudad de México, México. https://viruela.salud.gob.mx/docs/Manual_VE_Viruela_Simica.pdf.

[17] Petruccioli E., Sbarra S., Vita S., Salmi A., Cuzzi G., De Marco P., Matusali G., Navarra A., Pierelli L., Grifoni A. (2024). Characterization of the Monkeypox Virus [MPX]-Specific Immune Response in MPX-Cured Individuals Using Whole Blood to Monitor Memory Response. Vaccines.

[18] Mazzotta V., Lepri A.C., Matusali G., Cimini E., Piselli P., Aguglia C., Lanini S., Colavita F., Notari S., Oliva A. (2024). Immunogenicity and reactogenicity of modified vaccinia Ankara pre-exposure vaccination against mpox according to previous smallpox vaccine exposure and HIV infection: Prospective cohort study. EClinicalMedicine.

[19] Kaler J., Hussain A., Flores G., Kheiri S., Desrosiers D. (2022). Monkeypox: A comprehensive review of transmission, pathogenesis, and manifestation. Cureus.

[20] McCollum A.M., Damon I.K. (2014). Human monkeypox. Clin. Infect. Dis..

[21] Nkengurutse L., Otshudiema J.O., Kamwenubusa G., Diallo I., Nsavyimana O., Mbonicura J.C., Nkurunziza J.C., Cishahayo F., Niyongere D., Havyarimana B. (2025). Clinical predictors and determinants of mpox complications in hospitalized patients: A prospective cohort study from Burundi. Viruses.

[22] Meo S.A., Al-Masri A.A., Klonoff D.C., Alshahrani A.N., Al-Khlaiwi T. (2022). Comparison of biological, pharmacological characteristics, indications, contraindications and adverse effects of JYNNEOS and ACAM2000 monkeypox vaccines. Vaccines.

[23] Sanyaolu A., Marinkovic A., Okorie C., Prakash S., Haider N., Dixon Y., Izurieta R., Badaru O., Smith S. (2023). Review of the prevalence, diagnostics, and containment measures of the current mpox outbreak. World J. Clin. Cases.

[24] Rodríguez-Morales A., Bonilla-Aldana D.K., Cardona-Ospina J.A. (2025). Impact of the LGBT+ rights on reporting cases and deaths of Mpox globally: Relationships with the LGBT+ rights index during 2022–2024 epidemics. Int. J. Infect. Dis..

[25] Secretaría de Salud (2022). Dirección General de Epidemiología. Lineamientos para la Vigilancia por Laboratorio de Mpox. Versión 2.0. Ciudad de México, México. https://www.gob.mx/cms/uploads/attachment/file/984912/Lineamientos_mpox_03-2025.pdf.

[26] Li Y., Zhao H., Wilkins K., Hughes C., Damon I.K. (2010). Real-time PCR assays for the specific detection of monkeypox virus West African and Congo Basin strain DNA. J. Virol. Methods.

[27] Marie V., Gordon M.L. (2023). The (Re-)Emergence and Spread of Viral Zoonotic Disease: A Perfect Storm of Human Ingenuity and Stupidity. Viruses.

[28] Findlater A., Bogoch I.I. (2018). Human Mobility and the Global Spread of Infectious Diseases: A Focus on Air Travel. Trends Parasitol..

[29] Santaniello A., Perruolo G., Cristiano S., Agognon A.L., Cabaro S., Amato A., Dipineto L., Borrelli L., Formisano P., Fioretti A. (2023). SARS-CoV-2 Affects Both Humans and Animals: What Is the Potential Transmission Risk? A Literature Review. Microorganisms.

[30] Banuet-Martinez M., Yang Y., Jafari B., Kaur A., Butt Z.A., Chen H.H., Yanushkevich S., Moyles I.R., Heffernan J.M., Korosec C.S. (2023). Monkeypox: A review of epidemiological modelling studies and how modelling has led to mechanistic insight. Epidemiol. Infect..

[31] Alah M.A., Abdeen S., Tayar E., Bougmiza I. (2022). The story behind the first few cases of monkeypox infection in non-endemic countries, 2022. J. Infect. Public Health.

[32] Tuttle A., Hughes C.M., Dvorak M., Aeschleman L., Davidson W., Wilkins K., Gigante C., Satheshkumar P.S., Rao A.K., Minhaj F.S. (2024). Notes from the Field: Clade II Mpox Surveillance Update—United States, October 2023–April 2024. Morb. Mortal. Wkly. Rep..

[33] Scarpa F., Azzena I., Ciccozzi A., Branda F., Locci C., Perra M., Pascale N., Romano C., Ceccarelli G., Terrazzano G. (2024). Update of the Genetic Variability of Monkeypox Virus Clade IIb Lineage B.1. Microorganisms.

[34] Souza I.N., Pascom A.R.P., Spinelli M.F., Dias G.B., Barreira D., Miranda A.E. (2024). Demographic and clinical characteristics of people diagnosed with active sexually transmitted infections among monkeypox cases in Brazil: The 2022 outbreak. Rev. Inst. Med. Trop. Sao Paulo.

[35] Ward T., Christie R., Paton R.S., Cumming F., Overton C.E. (2022). Transmission dynamics of monkeypox in the United Kingdom: Contact tracing study. BMJ.

[36] Cuetos-Suárez D., Gan R.K., Cuetos-Suárez D., González P.A., Castro-Delgado R. (2024). A Review of Mpox Outbreak and Public Health Response in Spain. Risk Manag. Healthc. Policy.

[37] Bhulabhai M., Venugopal J., Plamondon M., Bergeron G., Cadieux G., Kancir J., Singal M., Twohig K., Zygmunt A., Schillberg E. (2025). Summary of the mpox outbreak in Canada, April 28–December 31, 2022. Can. Commun. Dis. Rep..

[38] Thornhill J.P., Gandhi M., Orkin C. (2024). Mpox: The Reemergence of an Old Disease and Inequities. Annu. Rev. Med..

[39] Secretaría de Salud (2024). Informes de la Vigilancia Epidemiológica de Mpox en México. Ciudad de México, México. https://www.gob.mx/salud/documentos/informes-de-la-vigilancia-epidemiologica-de-mpox-en-mexico.

[40] Gao S., Zeng Z., Zhai Y., Chen F., Feng X., Xu H., Kan W., Lu J., Zhou J., Chen Z. (2023). Driving effect of multiplex factors on Mpox in global high-risk region, implication for Mpox based on one health concept. One Health.

[41] Lu G., Chong Z., Xu E., Na C., Liu K., Chai L., Xia P., Yang K., Zhu G., Zhao J. (2025). Environmental, socioeconomic, and sociocultural drivers of monkeypox transmission in the Democratic Republic of the Congo: A One Health perspective. Infect. Dis. Poverty.

[42] Rosenberg E.S., Dorabawila V., Hart-Malloy R., Anderson B.J., Miranda W., O’Donnell T., Gonzalez C.J., Abrego M., DelBarba C., Tice C.J. (2023). Effectiveness of JYNNEOS Vaccine Against Diagnosed Mpox Infection—New York, 2022. MMWR Morb. Mortal. Wkly. Rep..

[43] Piccolo A.J.L., Chan J., Cohen G.M., Mgbako O., Pitts R.A., Postelnicu R., Wallach A., Mukherjee V. (2023). Critical Elements of an Mpox Vaccination Model at the Largest Public Health Hospital System in the United States. Vaccines.

[44] Kang H., Mahatanan R., Lee D., Locke S., Talbot E.A., Chan B.P. (2024). Understanding Barriers to Establishing Public JYNNEOS Mpox Vaccination Clinics in New Hampshire: Mpox vaccine clinic NH. Disaster Med. Public Health Prep..

[45] Gobierno de México (2024). Cofepris Autoriza Registro Sanitario a Vacuna Contra Mpox. Ciudad de México, México. https://www.gob.mx/cofepris/articulos/cofepris-autoriza-registro-sanitario-a-vacuna-contra-mpox?idiom=es.

[46] Danladi N.P., Agboola P., Olaniyi P., Eze S., Oladapo O., Obiwulu D., Akano O.S., Adeola O.A., Olawale K., Adiatu A.I. (2024). Challenges in Global Distribution and Equitable Access to Monkeypox Vaccines. Viruses.

[47] Tovani-Palone M.R., Doshi N., Pedersini P. (2023). Inequity in the global distribution of monkeypox vaccines. World J. Clin. Cases.

[48] Eurosurveillance editorial team (2024). Note from the editors: WHO declares mpox outbreak a public health emergency of international concern. Eurosurveillance.

